# Influence of the *White* Locus on the Courtship Behavior of *Drosophila* Males

**DOI:** 10.1371/journal.pone.0077904

**Published:** 2013-10-30

**Authors:** Dimitrije Krstic, Werner Boll, Markus Noll

**Affiliations:** Institute of Molecular Life Sciences, University of Zürich, Zürich, Switzerland; Queensland Brain Institute, Australia

## Abstract

Since its discovery by Morgan, the *Drosophila white* gene has become one of the most intensely studied genes and has been widely used as a genetic marker. Earlier reports that over- and misexpression of White protein in *Drosophila* males leads to male-male courtship implicated *white* in courtship control. While previous studies suggested that it is the mislocalization of White protein within cells that causes the courtship phenotype, we demonstrate here that also the lack of extra-retinal White can cause very similar behavioral changes. Moreover, we provide evidence that the lack of White function increases the sexual arousal of males in general, of which the enhanced male-male courtship might be an indirect effect. We further show that *white* mutant flies are not only optomotor blind but also dazzled by the over-flow of light in daylight. Implications of these findings for the proper interpretation of behavioral studies with *white* mutant flies are discussed.

## Introduction

The vinegar fly *Drosophila melanogaster* serves as an excellent model system to study the control of innate behaviors by the nervous system. The mating behavior of *Drosophila* consists of well-defined sequential steps that are usually repeated several times until copulation takes place [1]–[3]. While performing this elaborate courtship ritual, both partners receive a plethora of information in form of chemical and physical signals processed by their olfactory, gustatory, visual, auditory, and mechanosensory organs. These cues determine the courtship behavior and, through their integration in the brain, provide to both partners information about gender, conspecificity, receptivity, and even sexual fitness [4].

**Figure 1:**
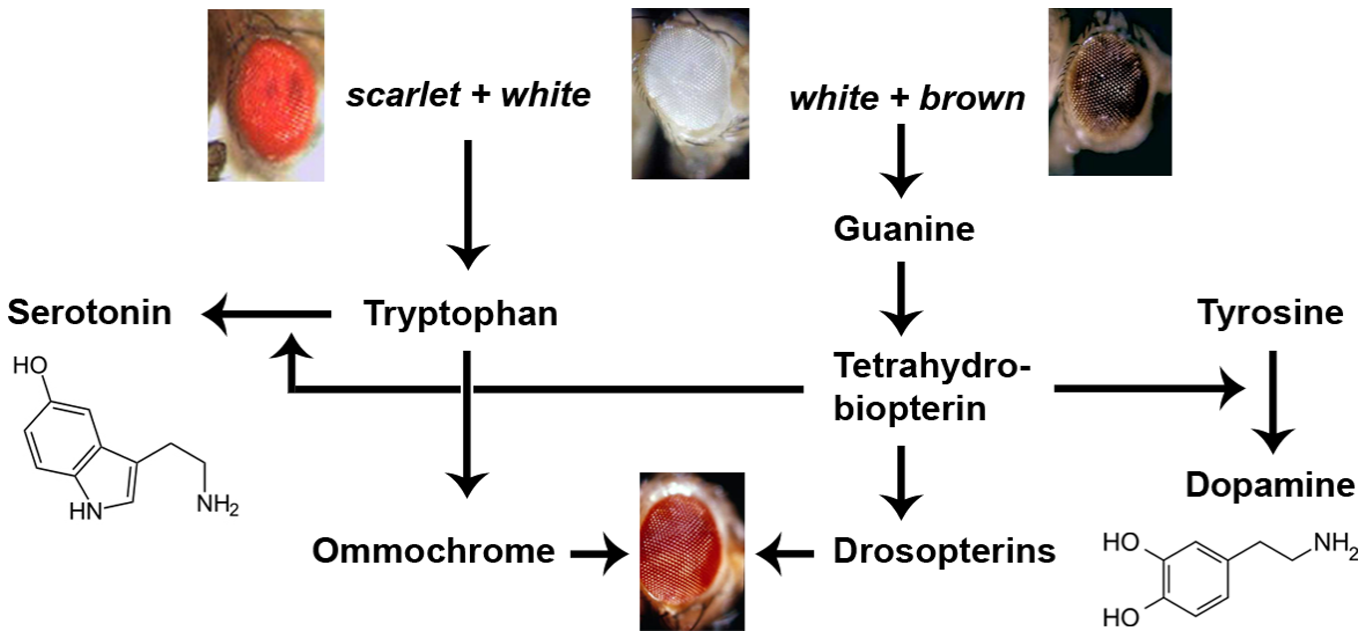
Role of White protein in the biosynthesis of *Drosophila* eye pigments and the neurotransmitters serotonin and dopamine. The White protein is an ABC transporter that, in combination with the Scarlet protein, transports tryptophan and, in combination with the Brown protein, guanine across the cell membrane into the cytoplasm [7]. Tryptophan is a precursor of the *Drosophila* Ommochrome pigment xanthommatin (brown) [6], but is also a precursor of the neurotransmitter serotonin [13], as illustrated on the left. Guanine is a precursor of tetrahydrobiopterin (BH_4_), which in turn is a precursor of most Drosopterins (red eye pigments of *Drosophila*) but also an essential cofactor in the conversion of tyrosine to dopamine, as indicated on the right, and of tryptophan to serotonin, as depicted on the left [11], [12].

The protein encoded by the *white* (*w*) gene of *Drosophila* is a transmembrane ABC transporter involved in the uptake of guanine and tryptophan, which are indispensable precursors in the synthesis of red (drosopterins) and brown (the ommochrome xanthommatin) *Drosophila* pigments [5]–[7] (Figure 1). The absence of pigments in the eye of flies without a functional *w* gene results in ommatidia without optical insulation. Hence the vision of such white-eyed flies is impaired, especially at high light intensities. These flies have a significantly reduced visual acuity [8] and show no response in optomotor tests [9]. Their photoreceptors receive about 19 times more light than those of wild-type flies [8], and their electroretinograms are abnormal [10]. Since guanine is further required for the synthesis of dopamine and serotonin [11], [12] and tryptophan is also a precursor of serotonin [13] (Figure 1), *w* mutants display altered levels and distributions of these neurotransmitters [14]. Accordingly, mutations affecting the *w* function have an impact on the neural control of various behaviors, independent of proper eyesight [15]–[19].

Specifically, *Drosophila* males that ectopically overexpress the *w* gene were shown to vigorously court each other and form courtship chains, a behavior called chaining [20], [21], which implied a function of *w* in the control of courtship behavior. Subsequent work suggested that increased male-male courtship results from overexpression-induced mislocalization of White protein within the cells that normally express it [22]. In apparent agreement with this hypothesis, *w* mutant males did not show enhanced male-male courtship and consequently did not chain [20]–[22].

Yet contrary to this hypothesis, we demonstrate here that the absence, or insufficient levels, of extra-retinal White protein causes enhanced male-male courtship, observed both in chaining and single-choice courtship assays. We further show that not only visual acuity of *w* mutant males is impaired, but that their behavior in daylight largely results from an overflow of light, which impeded the proper interpretation of earlier results on courtship behavior of *w* males [20]–[22]. In addition, we demonstrate that ‘rescue’ of *w* mutants by the standard *mini-white* gene does not rescue all behavioral aspects of wild-type behavior. Finally, we present results which suggest that the enhanced male-male courtship of *w* mutants is caused by a generally increased sexual arousal rather than the male’s inability to receive or process repellent sensory signals from the courted male.

## Materials and Methods

### Fly Stocks

The genotype of transgenic *Poxn-pRes* flies is *ΔXBs6*; *Poxn^ΔM22-B5^/Poxn^ΔM22-B5^* Δ*PBs96.2* [4], [23]. In these *Poxn^ΔM22-B5^* flies that are deficient for the *Poxn* gene, all *Poxn* functions are rescued by two *Poxn* transgenes, *ΔXBs6* and Δ*PBs96.2*, except those required for taste bristle development [4]. It should be noted that both *ΔXBs6* and Δ*PBs96.2* [23] include a *mini-white* marker gene in the pW6 vector [24]. The genotype of *mini-white* flies is *P{mini-white UAS-Poxn-6}* inserted on the second chromosome [25], [26]. Since two independent *mini-white* insertion lines produced comparable results, we present results from only one line. The *ninaB^360d^* mutant was kindly provided by William Pak. The *w^1118^* mutant and a translocation of the *white* locus to the Y chromosome, *Dp(1;Y)w^+^y^+^* (BL7060), were obtained from the Bloomington stock center.

### Courtship Assays

**Figure 2:**
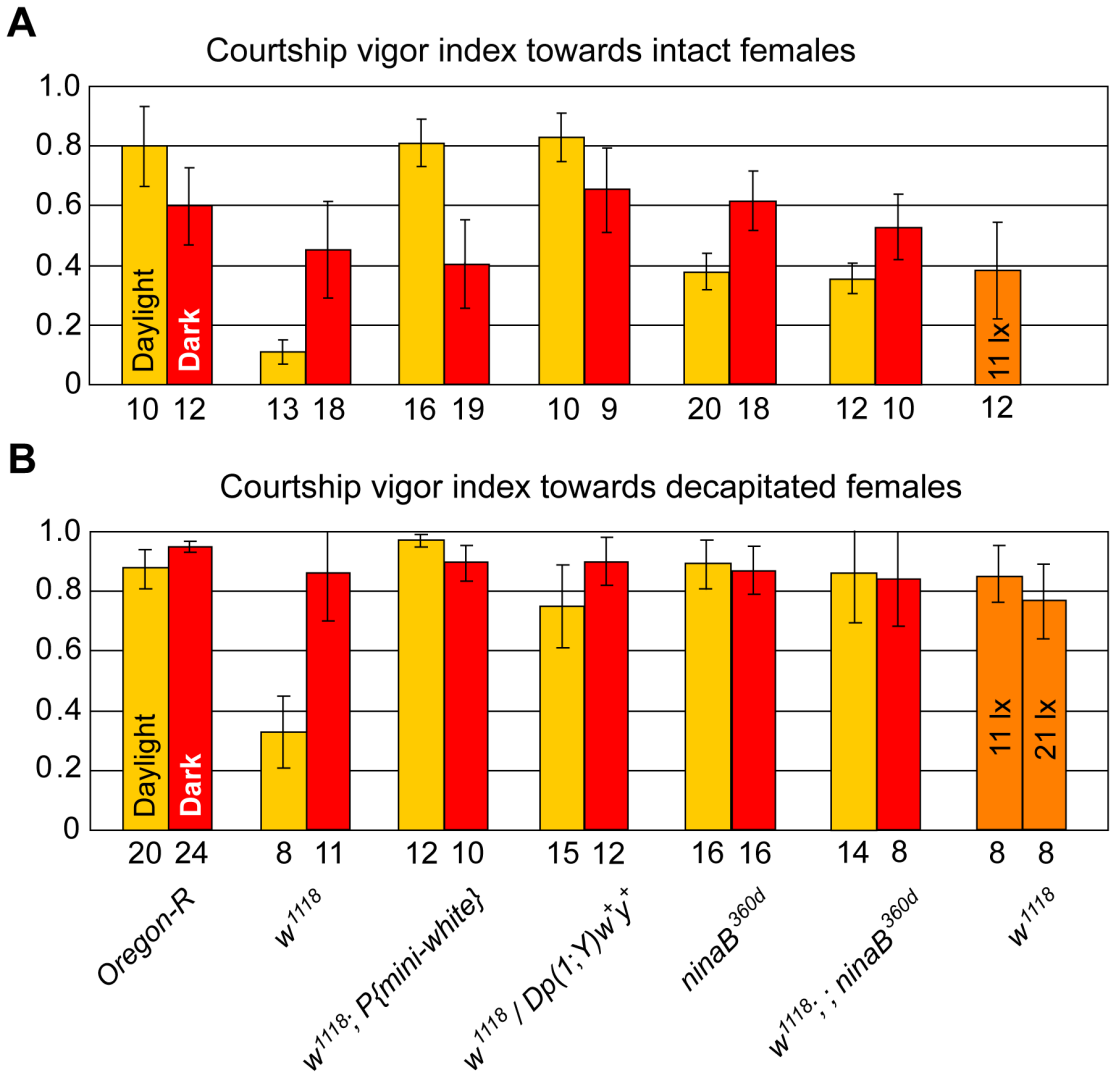
*w^1118^* flies are not only optomotor blind but also dazzled by light. Courtship vigor indices were measured in single-choice courtship assays with mature males of indicated genotypes and receptive (**A**) or decapitated (**B**) *Ore-R* virgin females in daylight (yellow columns), under dim red light (red columns), or under low-intensity light conditions (orange columns). The numbers below the columns indicate the number of couples observed that initiated courtship, and error bars always represent double standard errors of the mean. Data for *ninaB^360d^* males in panel A are from [4].

**Figure 3:**
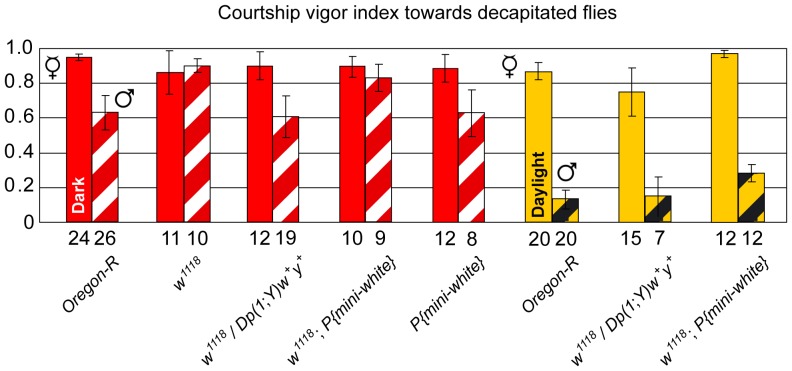
Lack of extra-retinal White function influences sexual orientation of males. Courtship vigor indices were measured in single-choice courtship assays with mature males of indicated genotypes and decapitated males (hatched columns) or decapitated females (filled columns) in the dark (red columns) or in daylight (yellow columns). The numbers below the columns indicate the number of males observed that initiated courtship. Data for *Ore-R* males are from [4].

**Figure 4:**
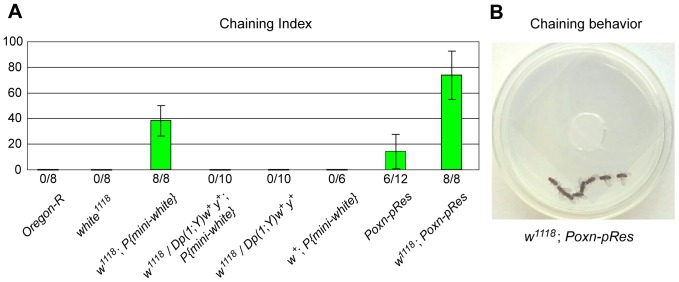
Chaining behavior of males is stimulated by a *w^1118^* background. (**A**) In addition to the average chaining indices for groups of eight males [30] of indicated genotypes, the number of groups for which chaining was observed over the total number of groups tested is shown. Error bars represent double standard errors. The result for *Poxn-pRes* males is from [4]. (**B**) Courtship chain of eight *w^1118^*; *Poxn-pRes* males. The picture was taken 10 min after eight mature, but sexually naïve, *w^1118^*; *Poxn-pRes* males were placed together into a small Petri dish.

**Figure 5:**
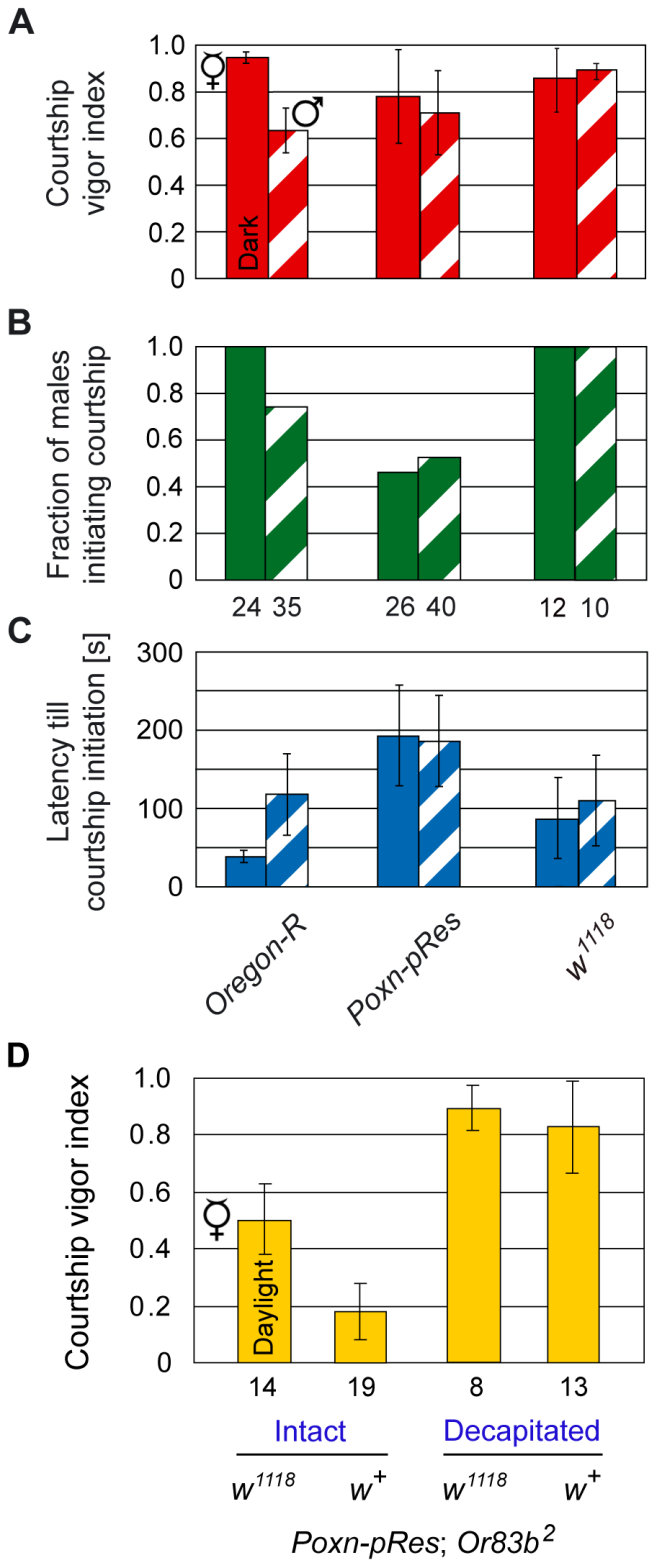
Increased sexual arousal of *w^1118^* males. (**A**–**C**) Male courtship parameters, the courtship vigor index (A), the fraction of males initiating courtship (B), and the average latency (in seconds) till courtship initiation (C), were measured in single-choice courtship assays with mature males of indicated genotypes and decapitated virgins (filled columns) or males (hatched columns) in the dark. (**D**) Courtship vigor indices of *Poxn-pRes*; *Or83b^2^* males, courting intact or decapitated females, were measured in single-choice courtship assays in daylight. *Poxn-pRes*; *Or83b^2^* males were either in a *w^1118^* or *w* ^+^ background. It should be noted that *w^1118^*; *Poxn-pRes*; *Or83b^2^* males carry two transgenes both of which include a *mini-white* marker gene (see Materials and Methods) that fully rescues pigmentation. Results for *Ore-R* and *Poxn-pRes* males in (A–C) and for *Poxn-pRes*; *Or83b^2^* males in (D) are from [4].

All courtship assays, manipulations of object flies, and fly husbandry were performed as described [4]. The courtship vigor index, cvi, is defined as fraction of time the male spent courting from courtship initiation, when first extending and vibrating a wing (love song), until copulation or the end of observation at 10 minutes [4]. Any of the following behaviors were scored as courting: wing vibration, tapping, licking, bending the abdomen, orienting, following with extended wings, and scanning. In daylight experiments with blind *ninaB^360d^* or *w^1118^* males, bilateral wing vibration as well as wing extension that was not directed towards the female was scored as courting since these males never displayed such behaviors in the absence of a female in the courtship chamber. Periods of courtship were measured by eye with a stop watch as the depth of the courtship chamber (9 mm) [4] does not permit the flies to be in focus of the camera during the entire observation period. In addition, flies spend most of their time on the wall of the chamber, which renders male courtship behaviors in movies (taken from above the chamber) difficult to interpret. The advantage of courtship chambers of the size used here has been emphasized previously [4]. Results shown in Figures 2–5 were supplemented, for comparison, with courtship data taken from [4], as specified in the legends to the figures. This is justified, as all courtship experiments presented here and in [4] were performed at the same time.

### Statistical Methods

Statistics was performed by the use of GraphPad Prism statistics software, applying the nonparametric Kruskal-Wallis test, which does not assume a Gaussian distribution of the results, followed by Dunn’s post test to compare all pairs of groups. Instead of indicating significance markers in figures, we provided p values in the text and used double standard error bars in figures to facilitate “comparison by eye” [27].

## Results

### Reduced Sexual Arousal of *w^1118^* Males in Daylight

In daylight, the sexual arousal, induced by the presence of a receptive virgin female, was dramatically reduced in *w^1118^* males compared to *Ore-R* males, as measured by their courtship vigor index cvi (Figure 2A, p<0.001; for cvi, see Material and Methods). This observation is consistent with the inability of *w* males to follow decamping females in daylight [4]. Accordingly, the low cvi of *w^1118^* males in daylight was raised again to that of wild-type males by a *mini-white* gene expressed in the eye or by a genomic *w* ^+^ gene translocated to the Y-chromosome (*P{mini-white}* or *Dp(1;Y)w^+^y^+^* in Figure 2A). In the dark, however, *w^1118^* males displayed wild-type behavior, as indicated by a cvi comparable to that of *Ore-R* males (Figure 2A, p = 0.14). This is explained by the fact that males, in search of a decamping female, switch in the dark to a scanning strategy [4], independent of their *w* gene activity. Similarly, the cvis of *w^1118^* males, rescued by a *mini-white* or genomic *w* ^+^ gene, did not differ significantly in the dark from the cvi of *Ore-R* males (Figure 2A, p = 0.11 or 0.51).

Astonishingly, the cvi of *w^1118^* males towards intact females in daylight was also considerably lower than that of blind *ninaB^360d^* males (Figure 2A, p<0.001), whose blindness is caused by a block in the synthesis of the retinal chromophore of rhodopsin [28], [29]. Moreover, unlike wild-type *Ore-R* and *ninaB^360d^* males that courted females, which had been decapitated to prevent them from decamping [4], very intensely in daylight, *w^1118^* males courted such females in daylight three times less vigorously (Figure 2B, p<0.001). Removal of the retinal chromophore by combining the *ninaB^360d^* with the *w^1118^* mutation enabled *w^1118^* males again to court both decapitated and intact females in daylight with a cvi comparable to that of *ninaB* ^360d^ males (cf. *w^1118^*; *ninaB^360d^* with *ninaB^360d^* males in Figure 2A, p = 0.61; and in Figure 2B, p = 0.49). In the dark, however, ‘blind’ single- or double-mutant males (*w^1118^*, *ninaB^360d^*, or *w^1118^*; *ninaB^360d^*) showed the same behavior as *Ore-R* males in both assays, i.e., with intact (Figure 2A) or decapitated females (Figure 2B). Finally, *w^1118^* males whose eye-pigmentation, and hence photoreceptor insulation, was rescued by means of *mini-white* or genomic *w* ^+^, courted decapitated females very vigorously, independent of light conditions (Figure 2B).

These observations led us to hypothesize that in daylight *w^1118^* males are not only lacking visual acuity and hence are optomotor blind, but that, in contrast to *ninaB^360d^* males, they are also dazzled by an excess of light because of the absence of photoreceptor insulation. To test this hypothesis, we evaluated the performance of *w^1118^* males towards females under low-light intensities, at illuminances of 11 lx and 21 lx, which are about 40 and 20 times lower than standard daylight laboratory conditions. Since these illuminances do not represent darkness for the flies, wild-type males do not switch to the scanning strategy after losing track of females, but use visual cues for their pursuit (Movie S1). Indeed, under low light intensities, *w^1118^* males courted decapitated females very intensely (orange bars at the far right in Figure 2B), and their cvi was comparable to that of *w^1118^* in complete darkness or when the chromophore of their rhodopsin had been removed by the *ninaB^360d^* mutation (Figure 2B). Similarly, intact females were courted by *w^1118^* males much more intensely at the reduced illuminance of 11 lx (Figure 2A, p<0.01) and to the same extent as by blind *ninaB^360d^* males in full daylight (Figure 2A), in good agreement with our hypothesis. Like wild-type males, *w^1118^* males were never observed scanning under these conditions. Therefore, it is the excess of light under standard daylight conditions that is responsible for the reduced sexual arousal of *w^1118^* males.

### Reduced Sexual Discrimination of *w^1118^* Males

We next examined the sexual orientation *w^1118^* males. As in daylight the overflow of light reduces the sexual arousal of *w^1118^* males, they were tested for their courtship preference in the dark and, to avoid the influence of repellent feedback signals from the courted male, with decapitated object males and females in single-choice courtship assays [4]. The cvi of *w^1118^* males towards decapitated males was enhanced as compared to that of *Ore-R* males (Figure 3, p<0.001) and equaled the cvi of *w^1118^* males towards decapitated females (Figure 3, p = 0.49). Strikingly, while supplementation of *w^1118^* males with a genomic *w* ^+^ gene reduced their elevated arousal towards decapitated males to the control level of *Ore-R* males (p = 0.22), their endowment with a *mini-white* gene clearly did not (Figure 3, p<0.01). As a control, males carrying the *mini-white* gene in a *w^+^* background behaved like *Ore-R* males (Figure 3). In addition, neither the inactivation of *w* nor its rescue by a *mini-white* or genomic *w* ^+^ gene affected the high cvi of males towards decapitated females, which was equal to that of *Ore-R* males (red filled columns in Figure 3, p = 0.31 for *w^1118^* vs. *Ore-R*, p = 0.10 for *w^1118^*; *P{mini-white}* vs. *Ore-R*, and p = 0.27 for *w^1118^*; *Dp(1*;*Y)w* ^+^ *y* ^+^ vs. *Ore-R*).

In daylight, *w^1118^* males, rescued by a genomic *w* ^+^ or *mini-white* gene, showed a clear preference toward decapitated females (Figure 3, p<0.001 in both cases). However, while *w^1118^* males, rescued with a genomic *w* ^+^ gene, displayed the same discrimination against decapitated males as *Ore-R* males (Figure 3, p = 0.46), *w^1118^* males, supplemented with *mini-white*, showed a significantly increased cvi towards decapitated males as compared to *Ore-R* males (Figure 3, p<0.01). Thus, *w^1118^* flies, rescued by a *mini-white* rather than a genomic *w* ^+^ gene, displayed a clear reduction of sexual discrimination in the dark as well in daylight.

### Lack of Extra-retinal White Protein in Males Induces Chaining

Since an effect of White overexpression on courtship behavior has previously been demonstrated for groups of flies [20], [21], we further examined *w^1118^* males in chaining assays, in which eight sexually naïve males were observed in daylight in a 10 mm x 35 mm Petri dish filled with a 6 mm agar layer [4]. Under these experimental conditions, *w^1118^* males, like wild-type *Ore-R* males, did not court each other, and thus no courtship chains were observed (Figure 4A), in agreement with previous observations [20]–[22]. However, it is important to emphasize that *w^1118^* males are not expected to display a chaining behavior because (i) they are optomotor blind and hence are unable to follow other flies using visual cues [4], and (ii) excess of light under standard daylight conditions decreases their sexual arousal (Figure 2). In contrast, *w^1118^* males rescued by a *mini-white* gene (*w^1118^*; *P{mini-white}*) started courting each other only a few minutes after they had been placed into the Petri dish, whereas *w^1118^* males rescued by a genomic *w* ^+^ gene (*w^1118^*; *Dp(1*;*Y)w* ^+^ *y* ^+^) did not chain (Figure 4A). The chaining indices of groups of *w^1118^*; *P{mini-white}* males, defined as the percentage of time three or more males form a chain during a 10-minute observation period [30], were not very high, and the chains consisted usually of only 3 or 4 males. This behavioral phenotype of *w^1118^* males carrying a *mini-white* gene was further rescued by a copy of the genomic *w* ^+^ gene or by replacing the *w^1118^* chromosome with a *w^+^* chromosome from *Ore-R* flies (Figure 4A). Thus, the missing or reduced discrimination between decapitated females and males in the dark (Figure 3) is similar to the difference in chaining behavior shown in Figure 4A. In both cases, this deficiency is evident in *w^1118^* males only when they are supplemented with a *mini-white* gene but not when they are endowed with a genomic *w* ^+^ gene. Since large parts of the control regions of the genomic *w* ^+^ gene have been deleted in the *mini-white* gene [26], this difference in behavior is interpreted in terms of a reduced or absent extra-retinal expression of White protein in *w^1118^* males supplemented with a *mini-white* gene.

As the chaining phenotype of *w^1118^* males rescued by a *mini-white* gene resembled that of taste-deficient *Poxn-pRes* males (Figure 4A) [4], we assumed that the *w^1118^* mutation may affect the processing of sex-specific gustatory information. This hypothesis was further consistent with the observation that *Poxn-pRes* males display equally high cvis towards decapitated males and females in the dark [4], like *w^1118^* males rescued by a *mini-white* gene (Figure 3). Yet, contrary to expectation and this hypothesis, double mutant *w^1118^*; *Poxn-pRes* males, which include two copies of a *mini-white* gene (Materials and Methods), displayed a considerably enhanced chaining behavior (Figure 4A,B). Moreover, all *w^1118^*; *Poxn-pRes* males participated in forming courtship chains almost all the time (Movie S2). We conclude that the reduced sexual discrimination in taste-deficient *Poxn-pRes* males is strongly enhanced by, and hence independent of, the lack of extra-retinal White protein in *w^1118^*; *Poxn-pRes* males.

### Increased Sexual Arousal of *w* Males Towards both Females and Males

Unlike *Ore-R* males, whose cvi shows a clear preference for decapitated females as compared to decapitated males [4], no such preference is observed with taste-deficient *Poxn-pRes* males or *w^1118^* males in the dark (Figure 5A). However, this lack of discrimination between decapitated females and males in the dark may have different reasons for taste-deficient *Poxn-pRes* males and *w^1118^* males deprived of vision. For, *Poxn-pRes* males are strongly inhibited from courtship initiation, as evident from the reduced fraction of males initiating courtship during the observation period (*Poxn-pRes* vs. *Ore-R* in Figure 5B, p<0.05 in both cases) and from a considerably extended time (latency) till courtship initiation towards females but not males (Figure 5C, p<0.01 and p = 0.07). By contrast, all *w^1118^* males initiate courtship towards both decapitated females and decapitated males (Figure 5B), while their latency till courtship initiation is slightly increased (p = 0.04) towards decapitated females, but unchanged (p = 0.82) towards decapitated males (*w^1118^* vs. *Ore-R* in Figure 5C). Thus, these two genetic manipulations, which both resulted in the loss of sexual preference, may have opposite effects on the state of sexual arousal in the courting male. While for a courting male the lack of gustatory perception reduced the attractiveness of a female and to some extent of a male [4], the absence of *w* functions increased its sexual excitability by a decapitated male in the dark. In daylight, on the other hand, lack of extra-retinal White also enhanced the sexual arousal of the courting male towards both decapitated males (Figure 3) and intact males, as reflected by the drastically augmented chaining of *w^1118^*; *P{mini-white}* and *w^1118^*; *Poxn-pRes* males (Figure 4 and Movie S2).

To test if lack of extra-retinal White increases the sexual arousal of males in general, i.e., also towards females, we took advantage of *Poxn-pRes*; *Or83b^2^* males which can neither smell nor taste [4]. In daylight, these males display a rather low cvi towards receptive females in single-choice courtship assays (Figure 5D) [4]. Yet, they court decapitated females very vigorously (Figure 5D). These observations indicate that vision alone does not provide sufficient sexual stimulation to maintain high levels of sexual arousal after the intact female decamps [4]. In agreement with a sexual arousal of *w* mutant males supplemented by *mini-white*, the lack of extra-retinal White protein increased the courtship vigor of *w^1118^*; *Poxn-pRes*; *Or83b^2^* males two- to three-fold towards intact receptive females in daylight (Figure 5D, p<0.001). The cvi towards decapitated females of these males, as compared to *Poxn-pRes*; *Or83b^2^* males, could not be significantly enhanced as the cvi of the latter was too close to 1 (Figure 5D).

## Discussion

### Influence of Light Over-flow on Courtship Behavior of *White* Males

We have shown in this study that in daylight *w^1118^* males court receptive virgin females with a cvi much lower than that of *Ore-R* males, as expected from the absence of proper vision on which a male depends to follow a decamping female (Figure 2A). However, in daylight these males also showed a low sexual arousal when facing decapitated females unable to decamp (Figure 2B). As expected, this striking behavioral phenotype of *w* null mutant males could be reverted to wild-type by rescue of the insulating eye-pigments with a *mini-white* or genomic *w* ^+^ gene. Surprisingly, however, the cvi of these *w^1118^* males was also (i) much lower than that of truly blind *ninaB^360d^* males, whose phototransduction is completely inhibited, and (ii) could be elevated to that of *ninaB^360d^* males by a drastic reduction of the light intensity during the test (Figure 2). As the NinaB protein is involved in the synthesis of retinoids, one might argue that the use of *ninaB* mutants has effects other than those caused by the block in chromophore synthesis of rhodopsin in photoreceptors. Such additional functions would also be impaired in *ninaB^360d^* mutants and might act upstream of extra-retinal White protein. Yet, it appears that such functions do not influence male courtship as no difference in courtship behavior of *ninaB^360d^* and *Ore-R* males, whose eyes were covered with black paint, were observed either in daylight or in the dark [4]. In the dark (dim red light), *w^1118^* males behaved like *Ore-R* males (Figure 2). Therefore, we concluded that in daylight *w* males are dazzled by the over-flow of light and that over-excitation of the photoreceptors suppresses their sexual arousal. In this context, it is remarkable that the sensitivity to the anesthetic halothane of *w^1118^* flies is increased in ambient light as compared to darkness, which suggests that light over-excitation affects the general state of arousal of *w* flies in daylight [31].

### Lack of Extra-retinal White in Males Increases their Sexual Arousal

In the absence of light over-excitation in the dark and of the rejective male feedback behavior prevented by decapitation, the preferred sexual orientation of males for females was completely abolished in *w^1118^* males (Figure 3). Wild-type sexual orientation was restored in these males by rescue with a genomic *w* ^+^ gene (*w^1118^*; *Dp(1*;*Y)w* ^+^ *y* ^+^) but not with a *mini-white* gene (Figure 3). In daylight, however, *w^1118^* males supplemented with a *mini-white* gene showed a clear preference for decapitated females, a behavior that is explained by the sexual discriminatory function of the restored vision in the courting male [4], but male-male courtship was elevated (Figure 3). Interestingly, *w* ^+^; *Dp(1*;*Y)w* ^+^ *y* ^+^ males displayed a slightly but significantly increased male-male courtship in daylight in this assay (data not shown). This increase in cvi probably results from the presence of two genomic *w* ^+^ copies in these males and the concomitant overexpression of White protein, in line with previous observations [20]–[22]. In the more complex chaining assay, *w^1118^* males supplemented with a *mini-white* gene engaged in male-male courtship and chaining (Figure 4). Note that in addition to the results presented in Figure 4, we tested six other *mini-white* lines in a *w^1118^* background and observed chaining behavior in all experiments (data not shown). Therefore, we argue that the *mini-white* gene used here and in numerous other studies, which is fully expressed in the eye, lacks enhancers of the wild-type *w* gene that express it in the CNS and/or PNS at levels sufficient to rescue all *w* functions and mutant behaviors. Thus, this extra-retinal *w* function is crucial to rescue all mutant behaviors. It follows that an extra-retinal function of White is important to control the sexual arousal in males. In addition and more generally, our findings caution against interpretations of results obtained in behavioral studies of *w* mutant flies rescued only by a *mini-white* gene. As the lack of extra-retinal White increases the sexual arousal of males also towards females, the observed male-male courtship and chaining behavior of *w^1118^* males, supplemented with *mini-white*, may be a secondary effect of an increased overall sexual excitability of these males. In addition, we would like to emphasize that the excessive and relentless chaining behavior of males (Movie S2) is observed only when in addition to the reduction or removal of extra-retinal White in *w^1118^*; *P{mini-white}* males, which stimulates their sexual arousal (Figure 5), gustatory reception, which inhibits male-male courtship [4], is abolished in *w^1118^*; *Poxn-pRes* males by the substitution of wild-type *Poxn* with the appropriate *Poxn* transgenes (Figure 4).

Since White protein is important for the cellular import of tryptophan, a precursor of serotonin [13], and serotonin levels are affected in *w* mutant flies [14], it is tempting to draw parallels between our observations and those made in rats, cats, and rabbits where the reduction of serotonin levels increased the sexual arousal of males [32]–[34]. These tried to mount not only females but also conspecific males. We suggest, therefore, that the *w* mutant phenotypes observed in our assays may at least in part be caused by lower levels of serotonin. In line with this suggestion, recent work showed that *w* mutant flies display an elevated phototactic personality and individual reaction to light stimuli, while this enhanced reaction is suppressed by *w*-dependent serotonin [35]. Therefore, it would be interesting to know whether and which behaviors other than courtship are affected by the lack of extra-retinal White and if these could also be induced by pharmacological reduction of serotonin levels.

It has been proposed that increased dopamine levels in *Drosophila* lead to an increase in general arousal, which results in an elevated behavioral responsiveness [36] and, more specifically, in an enhanced courtship vigor [37] as well as male-male courtship [38]. Surprisingly, however, a similar effect is achieved by a reduction of dopamine levels, which appears to decrease the threshold of arousal [36], thus rendering flies responsive to a lower intensity of stimuli. Although the effects of lower dopamine levels on courtship behavior still need to be confirmed, results with *w^1118^* males, whose serotonin as well as dopamine levels are reduced [14] (Figure 1), are consistent with such a bimodal function of dopamine.

### Models

The conspicuous mutant phenotype of the *w* gene was discovered by Morgan in 1910 and was the first reported *Drosophila* mutant [39]. Ever since, the *w* gene has been extensively used as a convenient genetic marker. Despite this fact, characterization of the *w* gene at the molecular level, other than determining its DNA sequence and, derived from it, that of its protein, has been hampered, mainly because the protein is expressed at very low levels. Thus, expression outside the eye, where its expression is obvious from its mutant phenotype, is not well documented. Hence, it is unclear where *w* is expressed in the central nervous system (CNS) and peripheral nervous system (PNS) of the adult fly. According to FlyBase, *w* is expressed at low to extremely low levels in the brain and CNS but no evidence for its expression in the PNS is known.

**Figure 6:**
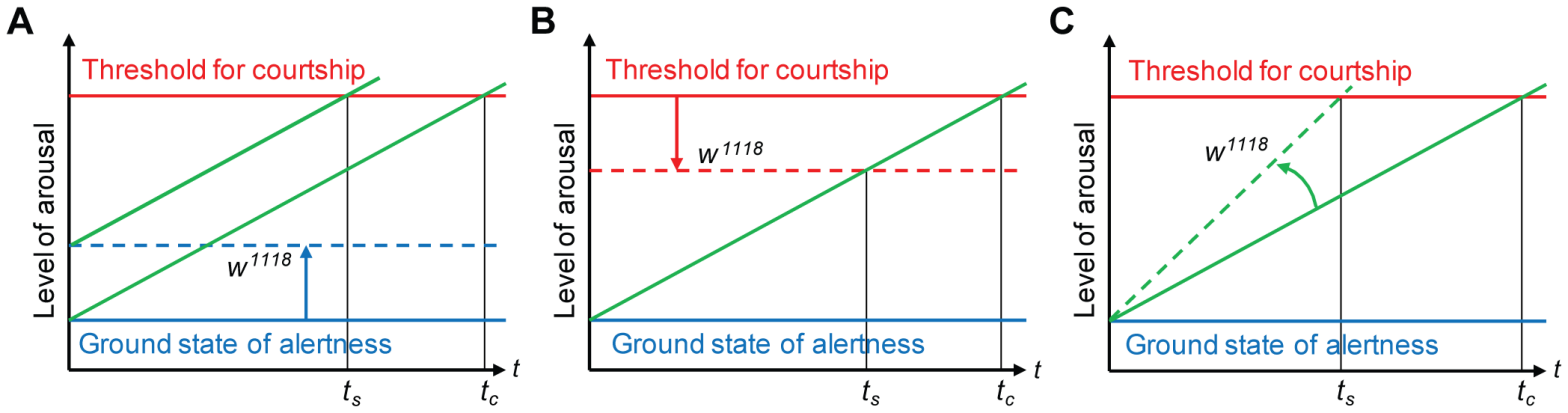
Effect of *white* mutation on sexual arousal of males. Models explaining possible effects of extra-retinal lack of White function on general sexual arousal of males. Increased sexual arousal of *white* males over time *t* may be caused by (**A**) a change in the general alertness from a ground state in wild-type flies (solid blue line) to an enhanced state of alertness in *w^1118^* mutants (broken blue line), resulting in a shortened, sensitized period of latency to sustained courtship, *t_s_*, as compared to the control period of latency, *t_c_*, or (**B**) a reduction of the threshold from its wild-type (solid red line) to a reduced *w^1118^* level (broken red line), which relevant stimuli need to reach to elicit sustained courtship, also resulting in a shortened, sensitized period of latency, *t_s_*. Since the processing of various sensory information by the CNS is not affected in either model, both changes can be taken to result from an enhanced sensitivity of the CNS to courtship stimuli. (**C**) Alternatively, *w^1118^* males may reach the threshold level for sustained courtship faster (broken green line in *w^1118^* mutants with slope greater than that of solid green line in wild-type flies) because of an altered processing or integration of courtship information by the CNS or an enhanced sensitivity to relevant stimuli, for example pheromones, of sensory neurons in the PNS. Also in this model, the augmented sexual arousal leads to a shortened, sensitized period of latency to sustained courtship, *t_s_*, because of a steeper slope of the broken versus the solid green line.

Therefore, based on the behavioral phenotypes of *w* mutants reported here, we suggest that the lack of White protein in the CNS, other than eye and ocelli, and perhaps in the PNS, increases the overall sexual arousal of males, leading them to indiscriminately court decapitated males and females in the dark, or to chain in daylight when placed in groups of males. This increased sexual arousal of *w* mutant males may result from an elevated alertness (Figure 6A), a reduction of the threshold for stimuli that elicit courtship (Figure 6B), or an enhanced sensitivity to pheromone stimuli (Figure 6C). While the first two models imply an augmented sensitivity for courtship in the CNS of courting *w^1118^* males, the third model suggests that these males exhibit an accelerated processing by the CNS of courtship-relevant information and perhaps an increased activity of PNS neurons that receive sensory stimuli for courtship initiation and maintenance. In each model, however, the interval of latency till sustained courtship is reduced from *t_c_* in wild-type to *t_s_* in *w^1118^* mutant flies (Figure 6). Although in reality a combination of these models may be relevant, further studies are required to discriminate between them. It should be emphasized that the effect of reduced or absent extra-retinal White protein on a male’s state of sexual arousal could be indirect. Thus, the absence or reduction of the extra-retinal *white* function might affect a network of the nervous system that is not involved in courtship control but whose state influences the circuitry regulating courtship behavior.

## Supporting Information

Movie S1 **Courtship assay clip of a wild-type** ***Ore-R*** **male after he initiated courtship.** Experiments were performed under illumination of 21 lx (first part) and 11 lx (second part). Under both conditions, the male orients towards and follows the female and does not switch to scanning behavior after the female decamps. The second part of the clip was recorded by the use of the “low-illumination mode” of the camera.
(AVI)

Movie S2 **A chaining assay video showing eight sexually naïve** ***w^1118^*** **;** ***Poxn-pRes*** **males in a small Petri dish.** All males participate in forming a courtship chain. The video was recorded 3–4 minutes after the males have been placed together.
(AVI)
